# Understanding Youth Suicide Risk Through Changes in Protective Strength Factors

**DOI:** 10.3390/bs16081282

**Published:** 2026-07-27

**Authors:** Saahoon Hong, Eric Kyere, Betty Walton, Hea-Won Kim

**Affiliations:** School of Social Work, Indiana University, 902 W. New York Street, Indianapolis, IN 46202, USA; ekyere@iu.edu (E.K.); beawalto@iu.edu (B.W.); heakim@iu.edu (H.-W.K.)

**Keywords:** adolescent suicide risk, protective strength factors, relationships, context

## Abstract

Adolescent suicide remains a critical public health concern, with rates continuing to rise globally. This study examined the role of strength development in mitigating suicide risk among youth receiving behavioral health services. Utilizing administrative data from a Midwestern state’s behavioral health authority, the study analyzed 391 adolescents aged 13–19 years (M = 16.34, SD = 1.51), including 244 girls (62.4%) and 147 boys (37.6%), who presented with recent suicidal ideation or behavior. Strength indicators included family strengths, interpersonal skills, optimism, educational support, vocational skills, talents and interests, spiritual or religious strengths, community involvement, relationship permanence, youth involvement in care, and natural supports. Latent Profile Transition Analyses identified two distinct strength groups: “usable strengths,” representing readily accessible protective factors, and “buildable strengths,” representing strengths requiring further development. Findings indicated that adolescents who transitioned from buildable to usable strengths experienced significantly lower suicide risk. Youth who maintained usable strengths across treatment also demonstrated substantially lower odds of suicide risk. Longer treatment duration was associated with lower suicide risk, whereas shorter treatment stays corresponded with elevated risk. Logistic regression analyses confirmed that maintaining or developing strengths related to social relationships, community connections, and supportive environments served as significant protective factors. These findings underscore the importance of integrating strength-based, relationship-centered, and community-informed approaches into behavioral health services to support resilience and reduce suicide risk among adolescents.

## 1. Introduction

For almost 60 years, adolescent suicide has been a critical public health concern across the United States (U.S.) and globally (Curtin, 2020). Despite recent declines in some regions, suicide remains a leading cause of death among children, adolescents, and young adults, with important variations across countries and demographic groups (Substance Abuse and Mental Health Services Administration [SAMHSA], 2025; World Health Organization [WHO], 2024). Globally, suicide ranks among the leading causes of death for individuals aged 15–29 years, while in the United States it remains the second leading cause of death among those aged 10–34 years (Centers for Disease Control and Prevention [CDC], 2023; World Health Organization [WHO], 2024). These trends underscore the urgency of identifying factors that protect youth from suicidal thoughts and behaviors.

Suicide risk is widely recognized as a complex phenomenon shaped by interrelated individual, relational, community, and societal influences (Substance Abuse and Mental Health Services Administration [SAMHSA], 2024a). Risk factors include previous suicide attempts, family history of suicidal behavior, mental illness, trauma exposure, substance use, chronic pain, impulsivity, self-injurious behavior, and environmental stressors (Ryan & Oquendo, 2020). Although substantial efforts have been devoted to identifying and predicting suicide risk, the accuracy of prediction remains limited, and prevention efforts have often relied on deficit-oriented frameworks that emphasize pathology and dysfunction (Su et al., 2023; Thangada & Kasoju, 2024).

A broader understanding of suicide emerges from sociological perspectives that emphasize the role of social relationships and community contexts. Durkheim (1897/2005) argued that suicide should not be understood solely as an individual act but as a social phenomenon influenced by the degree of social integration and regulation within families, communities, and institutions. His theory proposed that weakened social bonds and diminished social support increase vulnerability to suicide, whereas stronger connections provide individuals with meaning, belonging, and protection from distress. Contemporary scholars in critical suicidology have extended this perspective by arguing that suicide and suicidal behaviors exist within broader social, cultural, political, and historical contexts rather than arising exclusively from individual pathology (Button, 2016; Cover, 2020; Marsh, 2019; White & Morris, 2019). Together, these perspectives suggest that understanding suicide requires attention not only to risk factors but also to the social, relational, and developmental processes that foster resilience and protection. Contemporary resilience theory, particularly third-generation resilience research, conceptualizes resilience not as a fixed individual trait but as a dynamic, multisystem process involving interactions among individual capacities, family relationships, schools, communities, and broader social systems (Masten, 2014). From this perspective, resilience reflects the ability to maintain or regain adaptive functioning despite significant adversity and has been increasingly recognized as a protective factor against suicidal ideation, suicide attempts, and suicide-related mortality. Recent studies indicate that higher levels of resilience are associated with lower suicidal ideation, reduced psychological distress, and greater recovery following exposure to adverse life events, highlighting the importance of both individual and contextual protective processes in suicide prevention (Alonzo, 2022; Muyor-Rodríguez et al., 2021).

Complementing resilience theory, strengths-based and positive psychology frameworks emphasize the identification and cultivation of personal and environmental assets that support well-being and adaptive functioning (Peterson & Seligman, 2004). Character strengths such as hope, optimism, perseverance, social intelligence, gratitude, spirituality, and meaning have been identified as important protective factors against suicidal thoughts and behaviors (Peterson & Seligman, 2004; Sueki, 2021). Strengths-based approaches, therefore, extend beyond symptom reduction by focusing on the development and utilization of capacities that enable youth to cope effectively with adversity, build supportive relationships, and sustain engagement in meaningful activities. Within behavioral health systems, these strengths often include family support, interpersonal skills, school connectedness, community involvement, relationship permanence, youth participation in care, and natural supports that facilitate access to emotional, social, and practical resources (Devaney et al., 2023; Rasmus et al., 2019).

A growing body of research supports the importance of resilience- and strengths-based interventions in reducing suicide risk among adolescents. Programs such as Sources of Strength have demonstrated improvements in help-seeking behaviors, social connectedness, and perceptions of support from trusted adults, all of which are associated with lower suicide risk (Wyman et al., 2019). Community-driven interventions developed for Indigenous youth have similarly demonstrated that strengthening cultural identity, social support, and collective efficacy can enhance resilience and reduce vulnerability to suicidal behaviors (Allen et al., 2018). More broadly, systematic reviews indicate that adolescents who report stronger family cohesion, supportive peer relationships, school connectedness, optimism, and meaning in life are less likely to experience suicidal ideation and suicide attempts (Arango et al., 2024; Bakken et al., 2024; Marraccini & Brier, 2017).

Strengths-based interventions also provide opportunities to monitor how protective factors develop over time. Within the Child and Adolescent Needs and Strengths (CANS) framework, strengths are conceptualized along a developmental continuum. Usable strengths are those currently accessible and can be readily incorporated into treatment planning, whereas buildable strengths are present but require further development before they can function as effective protective resources (Lyons, 2022). Prior research has shown that youth who develop and sustain strengths over time demonstrate improved behavioral health outcomes and reduced functional impairment (Hong et al., 2021). However, relatively little is known about how transitions between buildable and usable strengths influence suicide risk trajectories among adolescents receiving behavioral health services. Examining these developmental transitions may provide important insights into how resilience-promoting processes and strengths-based interventions contribute to suicide prevention and recovery.

Rather than representing competing explanations, Durkheim’s sociological theory, resilience theory, and strengths-based perspectives provide complementary levels of explanation for understanding adolescent suicide risk. Durkheim’s theory emphasizes that suicide is influenced by the degree of social integration and regulation embedded within families, schools, communities, and other social institutions. Resilience theory extends this perspective by conceptualizing protective factors as dynamic developmental processes that emerge through interactions across multiple ecological systems rather than as fixed individual characteristics. The strengths-based framework operationalizes these processes within behavioral health practice by distinguishing between strengths that are immediately available to support intervention (usable strengths) and those that require further development before functioning as effective protective resources (buildable strengths). This integrated theoretical perspective suggests that changes in protective strengths reflect developmental changes in resilience and social connectedness rather than simply changes in individual functioning. Consequently, the central research question is not merely whether adolescents possess protective strengths, but whether they transition between qualitatively different configurations of strengths over time. Latent Profile Transition Analysis (LPTA) is therefore well suited to examine these developmental trajectories and to determine whether movement from buildable to usable strengths is associated with lower suicide risk.

## 2. Methods

### 2.1. Participants

**Figure 1:**
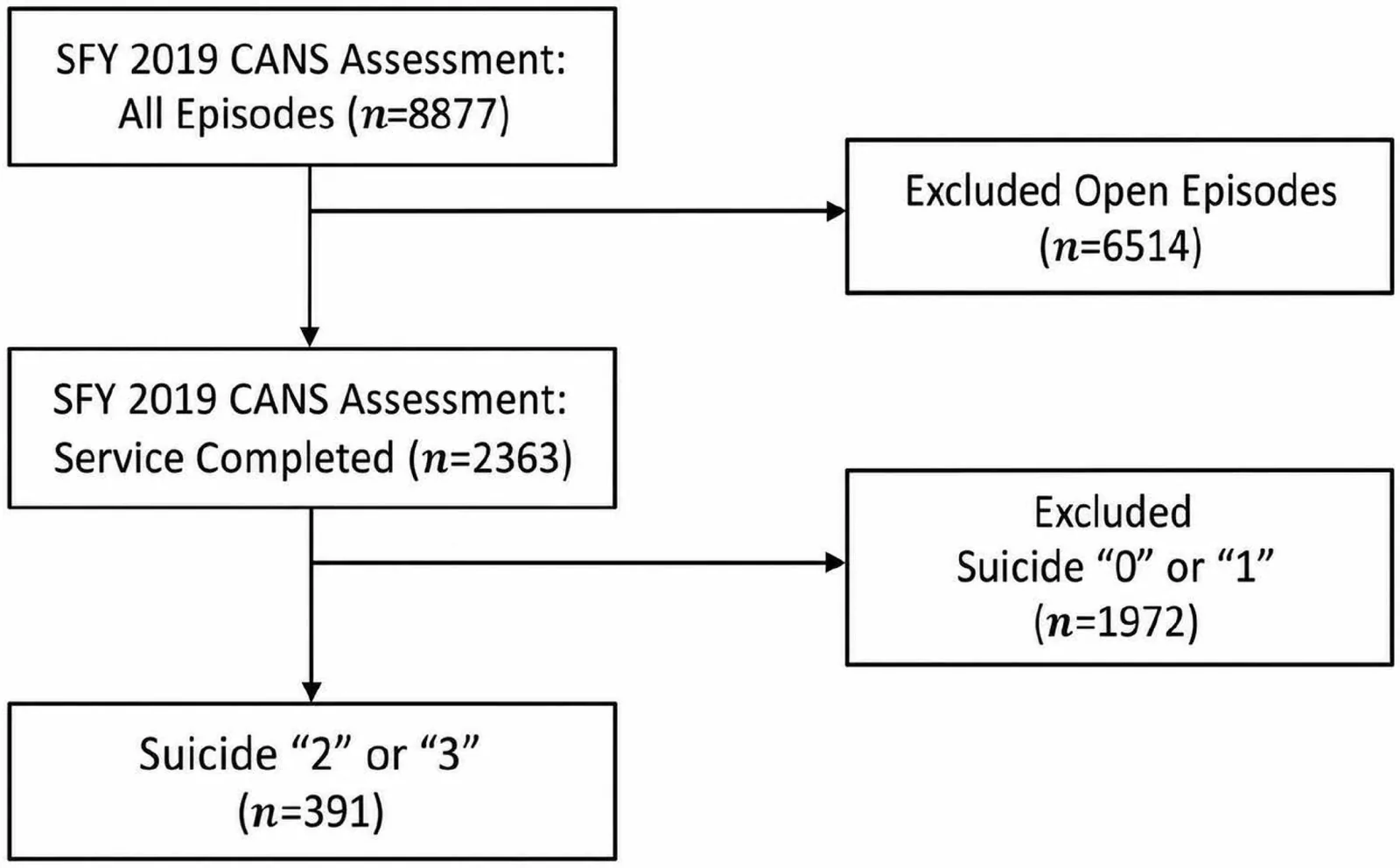
Flow diagram illustrating sample selection and exclusions.

This study used statewide administrative data from a behavioral health authority’s database in a Midwestern state, including demographic, assessment, and diagnostic information for service participants. The assessment provided person-level information about individuals’ strengths and needs. In this study, participants included 391 adolescents aged 13–19 who completed an episode of publicly funded behavioral health services during State Fiscal Year 2019. The sample was limited to adolescents who had recent (within the last 30 days) or current suicidal ideation or behavior that required safety planning and intervention. For this study, suicide risk was measured at the end of an episode of behavioral health care. Figure 1 illustrates the sample selection process leading to the final analytic sample. Exclusion criteria included open (incomplete) treatment episodes, episodes with initial Suicide Risk ratings below the study inclusion threshold, and cases with incomplete longitudinal assessment data that precluded estimation of latent class membership and, therefore, inclusion in the latent transition analysis.

Retrieved data included the initial and most recent CANS assessments for closed episodes of care with documented service completion, regardless of whether services began before State Fiscal Year 2019. As illustrated in Figure 1, of the 8877 total episodes identified, 6514 open episodes were excluded, leaving 2363 completed treatment episodes. Episodes with an initial Suicide Risk rating of 0 or 1 (*n* = 1972) were subsequently excluded. The final study sample consisted of 391 adolescents whose initial Suicide Risk ratings were 2 or 3, indicating clinically significant suicide risk requiring intervention. Although all 391 adolescents were included in the descriptive and regression analyses, the latent transition analysis was conducted on 388 participants with complete latent class membership information across both assessment waves. Three participants were excluded from the latent transition analysis because latent class membership could not be estimated due to incomplete assessment data across the two time points.

### 2.2. Measures

***Child and Adolescent Needs and Strengths*.** Strengths and suicide risk were measured using the Child and Adolescent Needs and Strengths (CANS; Lyons, 2022), a communimetric assessment designed to support treatment planning, service monitoring, and outcomes management in behavioral health systems. The CANS includes six domains comprising 64 items: youth strengths, life functioning, cultural factors, caregiver needs and resources, behavioral or emotional needs, and risk behaviors. This study focused on the Youth Strengths domain and the Suicide Risk item. The Youth Strengths domain consists of 11 indicators: family strengths, interpersonal skills, optimism, educational setting, vocational functioning, talents and interests, spiritual or religious strengths, community life, relationship permanence, youth involvement in care, and natural supports. Family strengths assess positive family relationships, communication, and emotional support. Interpersonal skills evaluate the youth’s ability to establish and maintain relationships with peers and adults. Optimism reflects positive expectations regarding the future. Educational setting measures the degree of support and engagement within the school environment. Vocational functioning assesses age-appropriate work-related or pre-vocational skills. Talents and interests capture hobbies, abilities, and activities that contribute to a positive sense of self. Spiritual or religious strengths assess access to spiritual support and meaning. Community life reflects connections to community organizations, activities, and institutions. Relationship permanence measures the stability of significant relationships. Youth involvement in care assesses participation in treatment planning and decision-making. Natural supports are unpaid individuals who provide social and emotional support within a youth’s environment.

Consistent with the communimetric principles of the CANS (Lyons, 2022), ratings of 0 and 1 were classified as usable strengths, reflecting strengths that are sufficiently developed to support treatment planning, whereas ratings of 2 and 3 were classified as buildable strengths, indicating strengths that require further development before functioning as effective protective resources. These cutoffs represent established clinical action thresholds within the CANS framework rather than empirically derived statistical categories and have been used in previous CANS-based longitudinal research (Hong et al., 2021).

***Suicide Risk***. Suicide risk was assessed using the Suicide Risk item from the CANS Risk Behaviors domain. Ratings range from 0 (no evidence of suicide risk) to 3 (dangerous or disabling suicide risk requiring immediate or intensive intervention). Consistent with the communimetric framework underlying the CANS (Lyons, 2022), ratings of 0 and 1 represent non-actionable levels of need, whereas ratings of 2 and 3 indicate clinically actionable suicide risk requiring intervention. Accordingly, discharge ratings were dichotomized into non-actionable suicide risk (0–1) and clinically actionable suicide risk (2–3) for the primary analyses. This classification reflects the clinical decision-making thresholds used within the CANS rather than a statistical recoding of the ordinal scale.

***Demographic and Clinical Characteristics*.** Demographic and clinical variables included age, gender, race, primary psychiatric diagnosis based on ICD-9 classifications, recommended intensity of care, Medicaid enrollment status, and treatment duration. Treatment duration was calculated as the total number of days between the initial and final assessments within the episode of care.

### 2.3. Procedure

The CANS assessment was implemented statewide by the behavioral health authority to support clinical decision-making, treatment planning, program management, and outcomes monitoring across publicly funded behavioral health services. During State Fiscal Year 2019, the CANS was utilized by 24 state-contracted community mental health centers serving children and adolescents throughout the state.

To ensure consistent administration and scoring, clinicians were required to complete standardized online training and maintain annual certification in the use of the CANS. In addition, supervisors and designated organizational coaches completed advanced training and certification to support local implementation, quality assurance, and ongoing fidelity to the assessment framework. Initial CANS assessments were completed at service entry, with reassessments conducted approximately every six months or at clinically significant transition points in accordance with statewide implementation guidelines. Consequently, the interval between the initial and final assessments varied across participants depending on treatment duration and clinical need.

Based on patterns of assessed needs, the state’s data management system generated recommendations regarding the intensity of care required to support treatment planning and service allocation. For the present study, de-identified assessment and service records were obtained from the statewide database under a data use agreement with the behavioral health authority. Data included demographic information, diagnostic characteristics, treatment history, and CANS assessments completed at intake and discharge. The initial assessment and the most recent assessment within a completed episode of care were extracted for analysis.

The Indiana University Institutional Review Board reviewed the study and determined it to be exempt from human participant oversight because all data were fully de-identified before analysis (IRB #1911059765). All procedures complied with applicable federal regulations governing the protection of human subjects (45 CFR 46) and institutional policies for secondary analysis of de-identified data. Reporting followed the Strengthening the Reporting of Observational Studies in Epidemiology (STROBE) guidelines.

### 2.4. Data Analysis

Latent profile analysis (LPA) was first performed to identify unobserved subgroups at two time points among youth in behavioral health treatment. The optimal number of latent profiles was determined by considering multiple statistical and substantive criteria, including the Akaike Information Criterion (AIC), Bayesian Information Criterion (BIC), adjusted BIC (ABIC), entropy, the Lo–Mendell–Rubin likelihood ratio test (LMR-LRT), class sizes, model parsimony, and the substantive interpretability of the resulting profiles. Because information criteria may continue to improve as additional classes are extracted, final model selection also emphasized theoretical coherence with the CANS framework and the clinical interpretability of the identified profiles. Eleven strength items at both the first and last assessments were the primary concerns of the unobserved subgroups. Using latent transition analysis (LTA), we examined different latent classes and their membership changes. In the LTA, transition probabilities based on the estimated model were the primary parameters of interest (e.g., the likelihood that youth classified in the usable strengths group (Class 1) at the initial assessment transitioned to the buildable strengths group (Class 2) at the final assessment).

Finally, logistic regression models were used to examine the association between strength-profile transitions and suicide risk at discharge while controlling for treatment duration, gender, race, and age. Prior to model estimation, multicollinearity among the predictor variables was assessed using tolerance statistics and variance inflation factors (VIFs). Conventional criteria were applied, with tolerance values below 0.20 or VIF values greater than 5 indicating potential multicollinearity. No evidence of problematic multicollinearity was observed. Following the latent transition analysis, participants were assigned to their most likely transition group based on posterior class membership probabilities (modal class assignment). The transition groups included usable → usable, representing youth who maintained usable strengths throughout treatment; usable → buildable, representing youth whose initially usable strengths became less accessible or required further development by discharge; buildable → usable, representing youth whose initially buildable strengths developed into usable strengths during treatment; and buildable → buildable, representing youth who maintained buildable strengths throughout treatment. The buildable → buildable group served as the reference category in the logistic regression analyses.

Following the latent transition analysis, participants were assigned to their most likely latent transition group based on posterior class membership probabilities (modal class assignment). The resulting transition groups—usable → usable, usable → buildable, buildable → usable, and buildable → buildable—were subsequently entered as predictors in logistic regression models examining suicide risk at discharge while controlling for treatment duration, gender, race, and age. The buildable → buildable group served as the reference category because it represented youth who maintained buildable strengths throughout treatment. Treatment duration was categorized into quartiles representing the shortest, short-to-medium, medium-to-long, and longest episodes of care. Gender was coded using females as the reference group, with males and youth whose gender identity was unavailable included as comparison groups. Race was coded using White youth as the reference group, with Black youth and youth from other racial backgrounds included as comparison groups. Age was treated as a continuous variable. All descriptive and logistic regression analyses were conducted using SPSS Version 26, whereas latent profile and latent transition analyses were performed using Mplus Version 7.

## 3. Results

The final analytic sample consisted of 391 adolescents aged 13 to 19 years (M = 16.34, SD = 1.51) who completed a publicly funded behavioral health treatment episode during State Fiscal Year 2019 and presented with recent or current suicide risk at intake. The sample included 244 females (62.4%) and 147 males (37.6%). The racial composition was 76.8% White, 13.1% Black, 5.9% other single race, 0.5% Native American, and 3.4% multiracial.

The mean interval between the initial and final assessments was 402.07 days (SD = 393.71), with assessment intervals ranging from 10 to 3119 days. To account for this substantial variability, treatment duration was categorized into quartiles and included as a covariate in the regression analyses. The most common primary psychiatric diagnoses were depressive disorders (21.5%), including major depressive disorder (single and recurrent episodes), conduct disorders (20.2%), attention-deficit/hyperactivity disorder (18.9%), and reaction to severe stress or adjustment disorder (14.6%). Based on assessed needs and recommended levels of care, supportive community-based services were the most frequently recommended service type (35.8%), followed by intensive community-based services (22.5%). Most participants were enrolled in Medicaid (76.5%). Additional demographic and clinical characteristics are presented in Table 1. Additional details are presented in Table 1.

**Figure 2:**
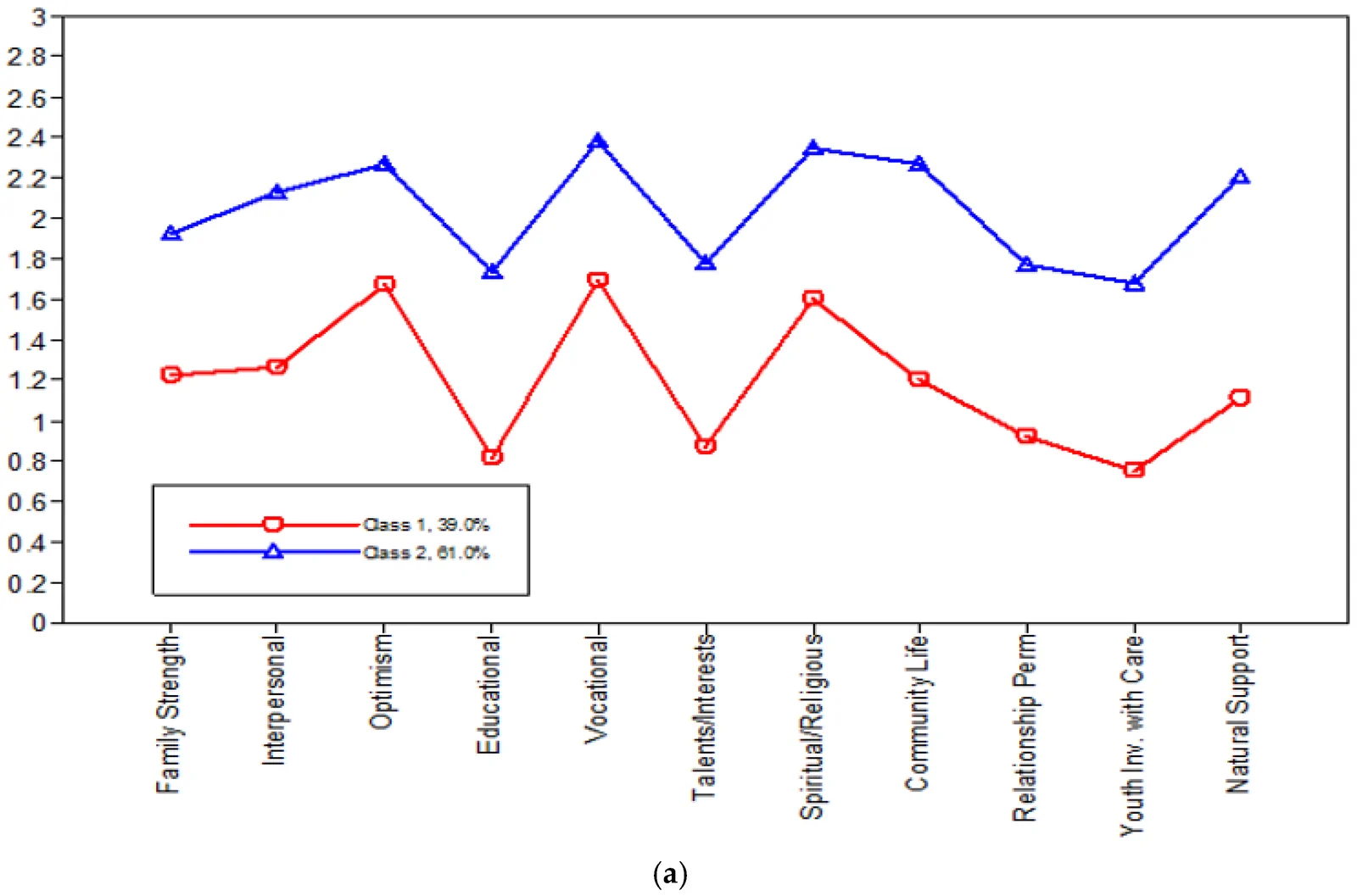

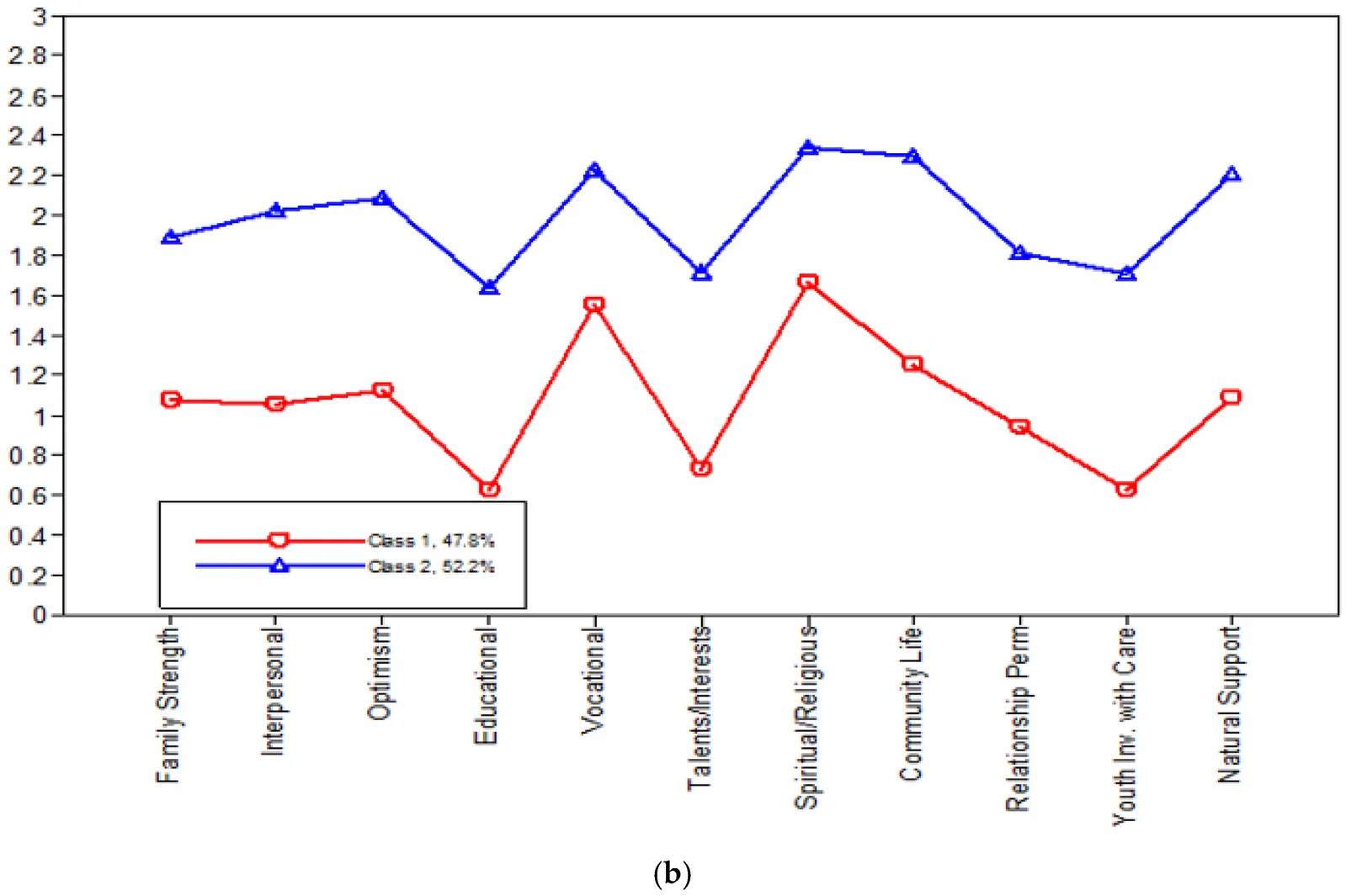
(**a**) Strengths in Two Classes at the Initial Assessment: Class Proportions. (**b**) Strengths in Two Classes at the Last Assessment: Class Proportions.

LPA was conducted to identify strength profiles at two time points: the initial assessment (Wave 1) and the final assessment (Wave 2). The results indicated that the best-fitting model at both waves consisted of two latent classes. At Wave 1, the two-class model showed the best fit according to several fit indices. Specifically, the AIC was 11,059.28, the BIC was 11,193.96, and the ABIC was 11,086.08. Additionally, the model demonstrated a significant likelihood ratio test (LRT = 545.21, *p* < 0.01) and an entropy value of 0.77, suggesting a clear separation between the two classes. These findings indicate the presence of two distinct strength profiles at the initial assessment. For the initial assessment, a usable strengths group (39%) and a buildable strengths class (61%) are presented in Figure 2a. The rate of a usable strengths group was 47.8%, and a buildable strengths group was 52.2% at the last assessment, shown in Figure 2b. At Wave 2, the two-class model demonstrated good classification quality, with an AIC of 14,973.08, a BIC of 15,107.76, an adjusted BIC of 14,999.88, a significant Lo–Mendell–Rubin likelihood ratio test (LRT = 694.60, *p* < 0.01), and an entropy value of 0.81, indicating a high degree of classification accuracy (Table 2). Although the three-class solution yielded lower information criteria, suggesting improved statistical fit, examination of the class structure indicated that the additional class primarily represented a subdivision of an existing profile rather than a qualitatively distinct pattern of strengths. Following established recommendations that latent profile selection should balance statistical fit with classification quality, parsimony, theoretical coherence, and substantive interpretability, the two-class solution was retained. This solution produced clinically meaningful profiles that were consistent with the established Child and Adolescent Needs and Strengths (CANS) framework, distinguishing between usable strengths and buildable strengths, while also facilitating parsimonious and interpretable longitudinal comparisons.

Overall, the distribution of strength ratings suggested considerable heterogeneity in youths’ access to protective resources at the beginning and end of treatment (Figure 2a,b).

**Figure 3:**
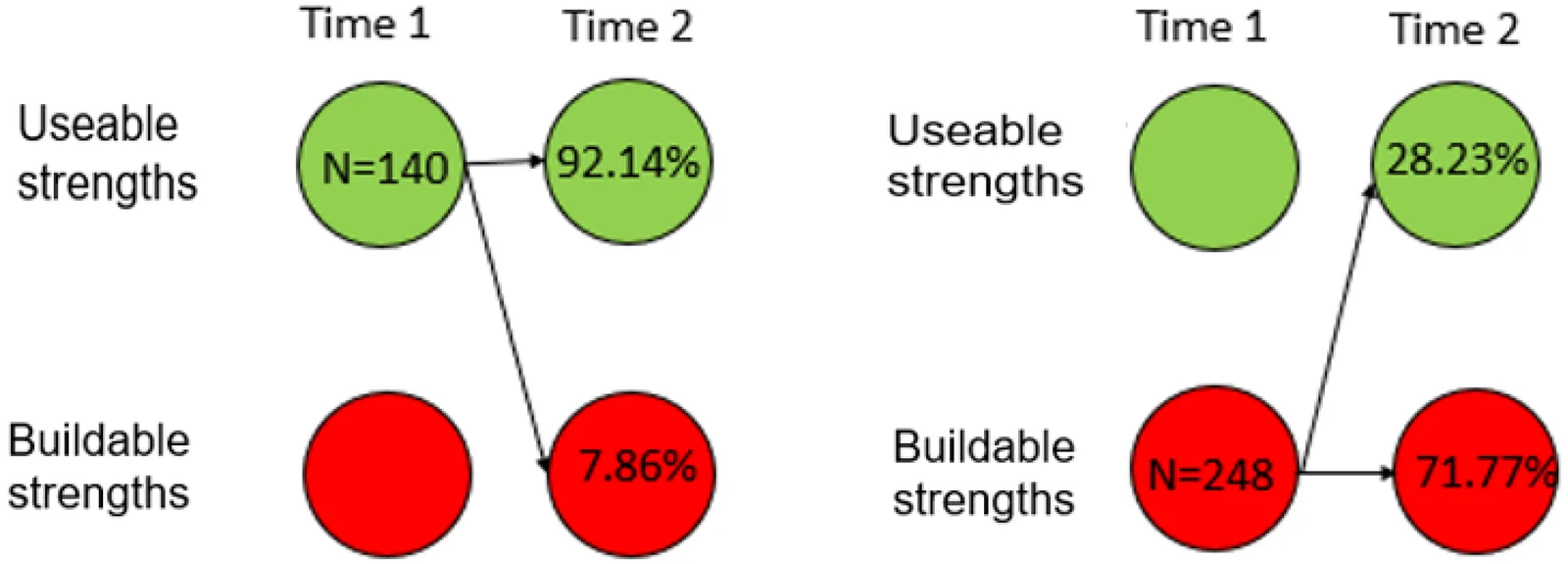
Final Class Counts and Proportions for the Latent Class Transition.

The two latent classes were interpreted as a usable strengths profile and a buildable strengths profile. At intake, 39.0% of youth were classified in the usable strengths profile and 61.0% in the buildable strengths profile. By discharge, the proportion of youth classified in the usable strengths profile increased to 47.8%, whereas 52.2% remained in the buildable strengths profile. Youth in the usable strengths profile demonstrated consistently stronger ratings across family strengths, interpersonal skills, optimism, educational support, community involvement, relationship permanence, and natural supports compared with youth in the buildable strengths profile. LTA estimated the probabilities of movement between the two strength profiles over time (see Figure 3). Among youth initially classified in the usable strengths profile, an estimated 92.14% remained in the usable strengths profile at discharge, whereas 7.86% transitioned to the buildable strengths profile. Among youth initially classified in the buildable strengths profile, 71.77% remained in the buildable strengths profile, while 28.23% transitioned to the usable strengths profile. These model-estimated transition probabilities indicate substantial stability among youth with established strengths and meaningful improvement among approximately one-third of youth who initially demonstrated buildable strengths.

The association between strength-profile transitions and suicide risk at discharge was examined using logistic regression, with the buildable → buildable group serving as the reference category (Table 3). Overall, the model demonstrated an acceptable fit to the data, as indicated by a non-significant Hosmer–Lemeshow goodness-of-fit test, χ^2^ (8, *N* = 391) = 7.37, *p* = 0.497. The model explained 23.1% of the variance in suicide risk according to the Cox and Snell pseudo-*R*^2^ and 34.1% according to the Nagelkerke pseudo-*R*^2^ (Cox & Snell *R*^2^ = 0.231; Nagelkerke *R*^2^ = 0.341), indicating moderate explanatory power.

Relative to youth who remained in the buildable → buildable group, those who maintained usable strengths throughout treatment (usable → usable) had significantly lower odds of suicide risk at discharge, OR = 0.35, 95% CI [0.19, 0.63], *p* < 0.001. Similarly, youth who transitioned from buildable → usable were significantly less likely to exhibit suicide risk at discharge, OR = 0.09, 95% CI [0.02, 0.30], *p* < 0.001. In contrast, the association between the usable → buildable transition and suicide risk did not reach statistical significance. Although the estimated odds ratio suggested a potentially protective association, the wide confidence interval indicates limited precision, likely due to the small number of adolescents in this transition group. Consequently, this finding should be interpreted cautiously and should not be viewed as evidence of the absence of an association. Rather, additional research with larger samples is needed to determine whether this transition is meaningfully associated with suicide risk.

Treatment duration was also significantly associated with suicide risk. Compared with youth in the longest treatment-duration quartile, those in the shortest-duration quartile were nearly 12 times more likely to exhibit suicide risk at discharge, OR = 11.79, 95% CI [4.51, 30.80], *p* < 0.001. Youth in the short-to-medium treatment-duration quartile were more than four times as likely to demonstrate suicide risk, OR = 4.27, 95% CI [1.60, 11.42], *p* < 0.001, whereas youth in the medium-to-long quartile did not differ significantly from those in the longest-duration quartile, OR = 1.91, 95% CI [0.67, 5.46], *p* = 0.23.

No significant associations were observed for race or gender, whereas age demonstrated a marginal negative association with suicide risk (OR = 0.84, 95% CI [0.70, 1.00], *p* = 0.05), suggesting that older adolescents tended to have lower odds of suicide risk at discharge. Although national epidemiological studies have consistently reported demographic differences in suicidal ideation, suicide attempts, and suicide mortality (Miranda-Mendizabal et al., 2019; Oh et al., 2025; Substance Abuse and Mental Health Services Administration [SAMHSA], 2022, 2023, 2024b), several factors may explain the absence of significant race and gender differences in the present study. First, the sample consisted exclusively of adolescents with clinically significant suicide risk at treatment entry, resulting in a relatively homogeneous high-risk population that may have reduced demographic variability in the outcome. Second, the outcome reflected clinician-rated suicide risk at discharge rather than suicide mortality or long-term suicidal behavior, outcomes that may exhibit different demographic patterns. Third, the multivariable analyses adjusted for treatment duration and longitudinal changes in protective strengths, factors that may be more directly associated with suicide risk than demographic characteristics and therefore may have attenuated demographic differences. Finally, the relatively small number of participants in some racial subgroups limited the statistical power to detect modest between-group differences. Accordingly, these findings should not be interpreted as evidence that demographic disparities in adolescent suicide risk are absent. Rather, they suggest that, within this clinical population, changes in protective strengths and treatment engagement were more strongly associated with clinician-rated suicide risk at discharge than demographic characteristics.

Collectively, the findings indicate that maintaining or developing usable strengths and remaining engaged in treatment for longer periods were associated with significantly lower suicide risk at discharge.

## 4. Discussion

The purpose of this study was to examine whether strength development and transitions in strength profiles were associated with suicide risk among adolescents receiving publicly funded behavioral health services. Consistent with resilience theory, strengths-based perspectives, and prior suicide prevention research, the findings suggest that adolescents who maintained usable strengths or transitioned from buildable strengths to usable strengths were significantly less likely to demonstrate suicide risk at discharge (Bakken et al., 2024; Hong et al., 2021). In addition, longer treatment duration was associated with lower suicide risk, indicating that both the availability of protective resources and sustained engagement in care may contribute to positive behavioral health outcomes.

The strongest predictors of lower suicide risk were maintaining usable strengths and developing buildable strengths into accessible protective resources. These findings are consistent with previous research demonstrating that improvements in strengths are associated with better behavioral health outcomes and reductions in functional impairment (Hong et al., 2021). Many of the strengths included in this study, such as family strengths, interpersonal skills, optimism, community involvement, relationship permanence, and natural supports, represent social and relational resources that can promote resilience, coping capacity, and adaptive functioning. Adolescents with stronger social connections may have greater access to emotional support, encouragement, problem-solving assistance, and a sense of belonging during periods of distress. These factors have been consistently associated with lower levels of suicidal ideation and behavior (Arango et al., 2024; Bakken et al., 2024).

The findings also support resilience-oriented conceptualizations of suicide prevention. Protective factors appear to function as developmental resources that can be strengthened over time rather than as fixed individual characteristics. Youth who transitioned from buildable strengths to usable strengths may have benefited from enhanced family engagement, improved interpersonal functioning, stronger school connections, and greater access to supportive community relationships. Such changes may increase resilience by helping youth manage adversity, regulate emotions, and maintain hope during periods of psychological distress. Since adolescents spend much of their time within family, school, and community environments, these systems likely play a critical role in strengthening protective factors and reducing suicide risk.

Longer treatment duration was significantly associated with lower suicide risk at discharge; however, this association should not be interpreted as evidence that longer treatment directly reduced suicide risk. Because treatment duration was observed within an administrative dataset rather than experimentally assigned, it may reflect differences in baseline clinical severity, family engagement, service accessibility, treatment adherence, or other unmeasured individual and system-level factors. Consequently, treatment duration should be viewed as an indicator of treatment engagement or service utilization rather than a causal measure of treatment effectiveness. Although sustained engagement in behavioral health services may provide greater opportunities to strengthen protective resources and therapeutic relationships, the present study cannot distinguish these potential mechanisms from selection effects or reverse causality. Active involvement of youth and caregivers in treatment planning may further reinforce treatment engagement and contribute to positive outcomes (Kothgassner et al., 2021; McCauley et al., 2018). In contrast, shorter treatment episodes may reflect barriers to sustained participation, including transportation difficulties, competing family responsibilities, housing instability, scheduling limitations, and limited access to services (Alvarez-Subiela et al., 2022; Branjerdporn et al., 2023; Liu & Wang, 2024).

The findings may also be understood through Durkheim’s sociological perspective, which emphasizes social integration and social regulation as protective mechanisms against suicide (Durkheim, 1897/2005). Youth with stronger family relationships, interpersonal skills, community involvement, and natural supports may experience greater belonging, purpose, and connection, thereby reducing vulnerability to suicidal thoughts and behaviors (Mueller et al., 2021; Wray et al., 2011). The stability of usable strengths observed among many participants suggests that social and relational resources may provide enduring protection against suicide risk. These findings are consistent with prior research using the CANS, which found that relational strengths, including family closeness, interpersonal skills, spirituality, community involvement, relationship permanence, natural supports, and optimism, were associated with lower suicide risk among youth receiving mental health services (Quiroga & Walton, 2014).

No significant associations were observed for race or gender, and age demonstrated only a marginal relationship with suicide risk. Although national surveys have reported demographic differences in suicidal thoughts and behaviors (Substance Abuse and Mental Health Services Administration [SAMHSA], 2022, 2023, 2024b), the present study focused on a clinical sample of youth already identified as experiencing elevated suicide risk. Furthermore, the outcome measure assessed suicide risk, including ideation, planning, intent, and attempts, rather than suicide mortality. This distinction may partially explain differences from epidemiological studies reporting higher suicide death rates among males (Miranda-Mendizabal et al., 2019; Oh et al., 2025).

The findings support the integration of strength-based assessment and intervention within adolescent behavioral health services. Routine monitoring of strengths may help clinicians identify protective resources that can be incorporated into treatment planning and suicide prevention efforts. Interventions that strengthen family relationships, interpersonal skills, optimism, school connectedness, community engagement, and natural supports may enhance resilience and reduce suicide risk. The findings also highlight the importance of sustaining youth engagement in treatment long enough for protective strengths to develop and stabilize. Behavioral health systems may therefore benefit from emphasizing both risk reduction and strength development as complementary goals of care.

Several limitations should be considered when interpreting these findings. First, the study utilized administrative data from a single Midwestern state’s publicly funded behavioral health system and included only adolescents who completed treatment episodes. Consequently, the findings may not generalize to youth who discontinue treatment prematurely, receive services in other healthcare systems, or reside in different geographic regions. In addition, the sample consisted exclusively of adolescents receiving behavioral health services and therefore may not represent youth in the general population. The administrative database also lacked information on ethnicity, LGBTQ+ identities, and other contextual factors that may influence suicide risk.

Second, because this study relied on secondary administrative data and an observational design, the findings should be interpreted as associations rather than causal relationships. In particular, treatment duration may reflect treatment engagement, baseline clinical severity, family support, or other unmeasured factors rather than a direct treatment effect. Future studies should employ causal inference approaches, such as survival analysis or propensity score methods, to better evaluate these relationships.

Third, both the predictor variables (Youth Strengths) and the outcome (Suicide Risk) were derived from the clinician-rated Child and Adolescent Needs and Strengths (CANS) assessment, introducing the possibility of common-method variance. Moreover, suicide risk was measured using a single CANS item at discharge without corroboration from standardized suicide assessments, medical records, crisis-service utilization, or longitudinal follow-up data. Future research should incorporate multiple informants and independent outcome measures to strengthen the validity of these findings.

Fourth, the classification of strengths into usable (0–1) and buildable (2–3) categories followed the established CANS action levels but may yield different latent profile structures than alternative operationalizations. In addition, several transition groups contained relatively few participants, resulting in wider confidence intervals and less precise parameter estimates. Future studies should evaluate alternative classification strategies and larger samples to improve the stability of subgroup estimates.

Finally, although the latent profile transition analysis was guided by statistical fit, parsimony, and theoretical interpretability, several methodological limitations remain. The Bootstrapped Likelihood Ratio Test (BLRT) was not estimated, longitudinal measurement invariance was not formally tested, and the distal outcome analysis used modal class assignment, which does not account for uncertainty in latent class membership. Furthermore, the data were collected during State Fiscal Year 2019, prior to the COVID-19 pandemic, and changes in adolescent mental health needs and behavioral health service delivery may limit the applicability of these findings to contemporary clinical populations. Future studies should replicate these analyses using more recent longitudinal datasets, incorporate more rigorous latent transition modeling procedures, and validate the findings across diverse populations and service systems.

## 5. Conclusions

This study advances understanding of how psychosocial strengths within youths’ ecological contexts function as protective mechanisms against suicide risk. Findings suggest that sustaining or developing usable strengths, particularly those reflecting relational, emotional, and community connections, significantly reduces the likelihood of suicide risk, reinforcing the centrality of strength-based engagement in behavioral health interventions. Longer treatment engagement was associated with lower suicide risk and the maintenance of protective strengths; however, this association should be interpreted cautiously given the observational nature of the study and the potential influence of unmeasured confounding factors. While sociodemographic factors such as race, gender, and age showed limited associations, the results emphasize the broader role of social integration and regulation, aligning with Durkheim’s framework that situates suicide within the context of social cohesion and belonging. Collectively, these insights call for a paradigm shift from individually focused, risk-driven interventions toward holistic, community-based, and relationally grounded models of care. Future research should integrate administrative data with Medicaid claims, crisis-service utilization records, and other longitudinal data sources to advance understanding of how systemic, environmental, and policy factors interact with the development of protective strengths to shape trajectories of resilience and suicide risk among diverse youth populations. In addition, these findings should be validated using more recent post-pandemic administrative data to determine whether changes in adolescent mental health needs and behavioral health service delivery have influenced longitudinal patterns of protective strengths and suicide risk.

## Figures and Tables

**Table 1:** Demographic Information (*N* = 391).

		*N*	%
Gender	Female	244	62.4
	Male	144	36.8
	NA	3	0.8
Race	Native American	1	0.5
	Black	40	13.1
	White	313	76.8
	Other single race	22	5.9
	More than one race	13	3.4
	NA	2	0.5
International Classification of Diseases (ICD) Diagnosis	Attention deficit disorder with hyperactivity	1	0.3
	Attention-deficit hyperactivity disorders	74	18.9
	Autistic disorders	5	1.3
	Bipolar affective disorder	9	2.3
	Conduct disorders	79	20.2
	Cyclothymic disorder	34	8.7
	Depressive disorder	1	0.3
	Impulse disorder	1	0.3
	Intermittent explosive disorder	2	0.5
	Major depressive affective disorder, recurrent episode, moderate	1	0.3
	Major depressive disorder, recurrent	57	14.6
	Major depressive disorder, single episode, mild	26	6.6
	Oppositional defiant disorder	1	0.3
	Other anxiety disorders	29	7.4
	Other paraphilias	1	0.3
	Other Specified episodic mood disorder	1	0.3
	Phobic anxiety disorders	1	0.3
	Reaction to severe stress, and adjustment disorders	57	14.6
	Reactive attachment disorder of childhood	7	1.8
	Schizoaffective disorder, depressive type	2	0.5
	Schizophrenia	1	0.3
	Unspecified episodic mood disorder	1	0.3
Recommended Level of Care	Outpatient	2	0.5
	Outpatient with limited case management	48	12.3
	Supportive community services	140	35.8
	Intensive community—based services	88	22.5
	Intensive home and community—based services	67	17.1
	High Intensity Services	46	11.8
Medicaid	Yes	299	76.5
	No	92	23.5
Days in the treatment	First quartile (Q1)	98	25.1
	Second quartile (Q2)	98	25.1
	Third quartile (Q3)	98	25.1
	Fourth quartile (Q4)	97	24.8
	**Mean**	**SD**	**Min/Max**
Days in the treatment	402.07	393.71	10/3119
Age	16.34	1.51	13.07/19.45

*Note*. NA = Missing information.

**Table 2:** Latent Profile Analysis: Model Fit Information.

	# of Classes			
	1	2	3	4
# of free parameters	22	34	46	58
Time 1: Initial				
Loglikelihood	−5772.06	−5495.64	−5433.09	−5389.99
AIC	11,588.12	11,059.28	10,958.18	10,895.98
BIC	11,675.26	11,193.96	11,140.38	11,125.72
ABIC	11,605.46	11,086.08	10,994.43	10,941.69
Entropy		0.77	0.74	0.79
LRT		545.21 **	123.38 *	85.01
Time 2: Last				
Loglikelihood	−7804.70	−7452.54	−6470.94	−6391.21
AIC	15,653.40	14,973.08	13,033.88	12,898.42
BIC	15,740.54	15,107.76	13,216.09	13,128.16
ABIC	15,670.73	14,999.88	13,070.13	12,944.13
Entropy		0.81	0.88	0.82
LRT		694.60 **	698.49	157.70

* *p* < 0.05; ** *p* < 0.01.

**Table 3:** Logistic Regression Model: Suicide Risk.

	B	S.E.	Wald	df	Sig.	Exp (B)	95% CI	
							Lower	Upper
Transition			24.82	3	0.00			
useable -> useable	−1.05	0.31	11.89	1	0.00	0.35	0.19	0.63
useable -> buildable	−1.90	1.11	2.93	1	0.09	0.15	0.02	1.32
buildable -> useable	−2.46	0.64	14.96	1	0.00	0.09	0.02	0.30
Days in the treatment			37.34	3	0.00			
Q1	2.47	0.49	25.37	1	0.00	11.79	4.51	30.80
Q2	1.45	0.50	8.35	1	0.00	4.27	1.60	11.42
Q3	0.65	0.54	1.46	1	0.23	1.91	0.67	5.46
Female			2.48	2	0.29			
Male	−0.46	0.29	2.48	1	0.12	0.63	0.35	1.12
Other	−19.99	28,126.07	0.00	1	1.00	0.00	0.00	
White			1.07	2	0.59			
Black	0.44	0.44	0.97	1	0.32	1.55	0.65	3.70
Other	−0.10	0.50	0.04	1	0.84	0.90	0.34	2.43
Age	−0.18	0.09	3.68	1	0.05	0.84	0.70	1.00
Constant	1.19	1.60	0.56	1	0.46	3.30		

## Data Availability

The data supporting the findings of this study are maintained by the Indiana Division of Mental Health and Addiction (DMHA). Access to these data is restricted and governed by DMHA data-use agreements; therefore, the dataset is not publicly available. Data could be made available from the authors upon reasonable request, provided formal approval is obtained from the Indiana DMHA.
